# Artificial intelligence in the care of children and adolescents with chronic diseases: a systematic review

**DOI:** 10.1007/s00431-024-05846-3

**Published:** 2024-12-14

**Authors:** Janna-Lina Kerth, Maurus Hagemeister, Anne C. Bischops, Lisa Reinhart, Juergen Dukart, Bert Heinrichs, Simon B. Eickhoff, Thomas Meissner

**Affiliations:** 1 Dept. of General Pediatrics, Pediatric Cardiology and Neonatology, Medical Faculty, University Children’s Hospital Düsseldorf, Heinrich Heine University, Moorenstr. 5, 40227 Düsseldorf, Germany; 2 Institute of Neuroscience and Medicine, Brain & Behaviour (INM-7), Research Centre Jülich, Jülich, Germany; 3 Institute of Systems Neuroscience, Medical Faculty & University Hospital Düsseldorf, Heinrich Heine University Düsseldorf, Düsseldorf, Germany; 4 Institute for Science and Ethics, University Bonn, Bonn, Germany

**Keywords:** Artificial intelligence, Machine learning, Chronically Ill children and adolescents, Pediatrics

## Abstract

The integration of artificial intelligence (AI) and machine learning (ML) has shown potential for various applications in the medical field, particularly for diagnosing and managing chronic diseases among children and adolescents. This systematic review aims to comprehensively analyze and synthesize research on the use of AI for monitoring, guiding, and assisting pediatric patients with chronic diseases. Five major electronic databases were searched (Medline, Scopus, PsycINFO, ACM, Web of Science), along with manual searches of gray literature, personal archives, and reference lists of relevant papers. All original studies as well as conference abstracts and proceedings, focusing on AI applications for pediatric chronic disease care were included. Thirty-one studies met the inclusion criteria. We extracted AI method used, study design, population, intervention, and main results. Two researchers independently extracted data and resolved discrepancies through discussion. AI applications are diverse, encompassing, e.g., disease classification, outcome prediction, or decision support. AI generally performed well, though most models were tested on retrospective data. AI-based tools have shown promise in mental health analysis, e.g., by using speech sampling or social media data to predict therapy outcomes for various chronic conditions.

*Conclusions*: While AI holds potential in pediatric chronic disease care, most reviewed studies are small-scale research projects. Prospective clinical implementations are needed to validate its effectiveness in real-world scenarios. Ethical considerations, cultural influences, and stakeholder attitudes should be integrated into future research.

**Table:** 

**What is known:** *• Artificial Intelligence (AI) will play a more dominant role in medicine and healthcare in the future and many applications are already being developed.*	
**What is new:** *• Our review provides an overview on how AI-driven systems might be able to support children and adolescents with chronic illnesses.* • *While many applications are being researched, few have been tested on real-world, prospective, clinical data.*

**Supplementary Information:**

The online version contains supplementary material available at 10.1007/s00431-024-05846-3.

## Introduction

Since the advent of research on artificial intelligence (AI) and its subfield machine learning (ML), medicine has been viewed as a field with ample possibilities of its applications [1]. Much of the research conducted involved diagnostic imaging in radiology and pathology, as well as tools to assist physicians and medical staff in making diagnoses. As AI can process and analyze large quantities of data in a short amount of time, it might play a crucial role in establishing personalized care and targeted treatments [2]. A systematic review by Rahimi et al. found that AI can be useful in primary healthcare settings with limited available resources for treatment planning and patient education as well as self-management of chronic diseases, allowing patients to track their symptoms and get personalized recommendations and health alerts [3]. This is particularly important considering the anticipated shortage of physicians and care especially in rural areas [4].

However, there are some particularities to consider when minors are involved. They might not be able to participate in decisions concerning the use of their healthcare data and agreement to the use of AI-based applications [5]. Young patients are oftentimes much more adept at the use of digital applications, generating larger amounts of non-traditional medical data in addition to electronic health care records or vital sign monitoring [6].

Considering this, there might be great potential in using AI-based applications not only in diagnostics, but also in day-to-day care for pediatric patients with chronic diseases. In this systematic review, we provide a comprehensive overview of research being conducted on the use of AI for monitoring, guiding, and assisting children and adolescents with chronic diseases.

## Methods

### Design

This systematic review followed the *Preferred Reporting Items for Systematic Reviews and Meta-Analyses* (PRISMA) guidelines [7].

It was registered with the International Prospective Register of Systematic Reviews (PROSPERO; registration ID: CRD42022344316).

### Search strategy

Five electronic databases (Medline, Scopus, PsycINFO, ACM, and Web of Science) were searched using the terms (artificial intelligence*) AND (chronic disease*) AND ((child*) OR (adolescen*)). The first 1000 results on Google and on Google Scholar were manually searched for gray literature and complemented by the search of personal archives and manual screening of reference lists. The searches were conducted on 07 July 2022 and updated on 06 February 2024. There were no restrictions regarding year of publication and no restriction for language other than using English search terms (see Appendix 2).

### Study selection

#### Inclusion criteria

All original studies (i.e., randomized controlled trials, non-controlled trials, qualitative studies, case reports) on the use of artificial intelligence in monitoring, and assisting children and adolescents with chronic diseases. For a more comprehensive overview, conference abstracts and proceedings were included.

#### Exclusion criteria

Non-AI-based digital health interventions and descriptions of diagnostic tools and processes. All non-original records such as commentaries, editorials, reviews, and position papers.

### Screening process

Records were imported into EndNote X9 (Clarivate Analytics, Philadelphia, PA, USA). A pilot screening was conducted jointly by two researchers for ten records for clarification and specification of inclusion and exclusion criteria.

After removal of duplicates, titles and abstracts were screened by two researchers individually. If the available information did not suffice or the decision was not unanimous, full text was assessed. Full texts were assessed for eligibility, and disagreement between the researchers was resolved through discussion with a third researcher.

### Data extraction

Data were extracted for year of publication, journal, country of study, language of publication, AI method used, study design, population, methods and/or intervention, and main results. A short summary was included. Piloting included five full texts and led to refinements of categories and documentation requirements. Data extraction was carried out independently by two researchers.

### Risk of bias assessment

Risk of bias was assessed using the Joanna Briggs Institute’s checklists for critical appraisal [8]. Bias was assessed individually by two researchers after piloting. Discrepancies were resolved through debate without the need to involve another researcher. No inclusion or exclusion decisions were to be based on this assessment.

### Data synthesis

Data were synthesized quantitatively when reasonable and meaningful, but as there was a broad variation in reporting of the results as well as research methodology, pooling of data was neither feasible nor adequate. We chose a narrative, qualitative approach to summarize findings. Studies were characterized as described above, commonalities described, and studies grouped accordingly.

## Results

**Fig. 1:**
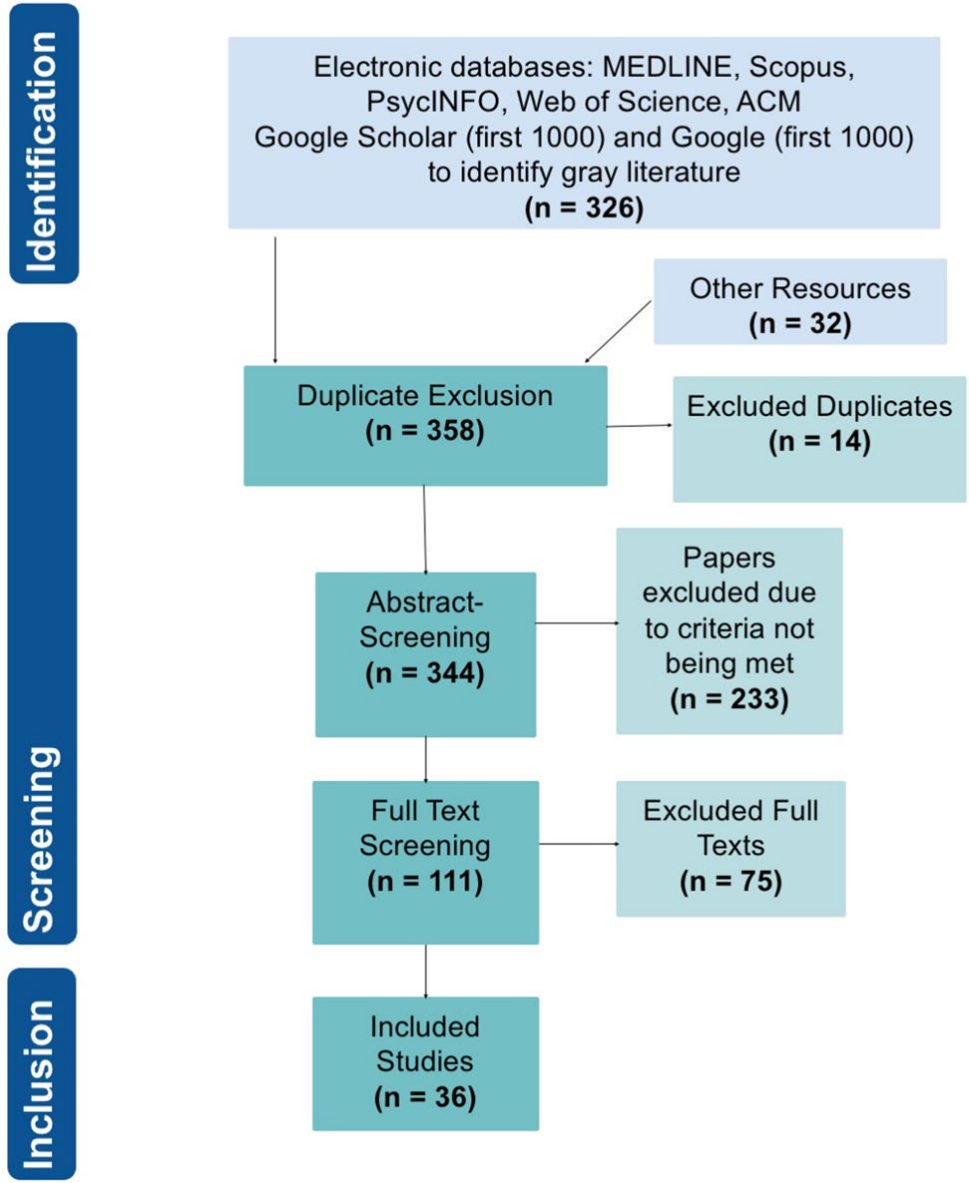
PRISMA flowchart depicting the literature screening process

**Fig. 2:**
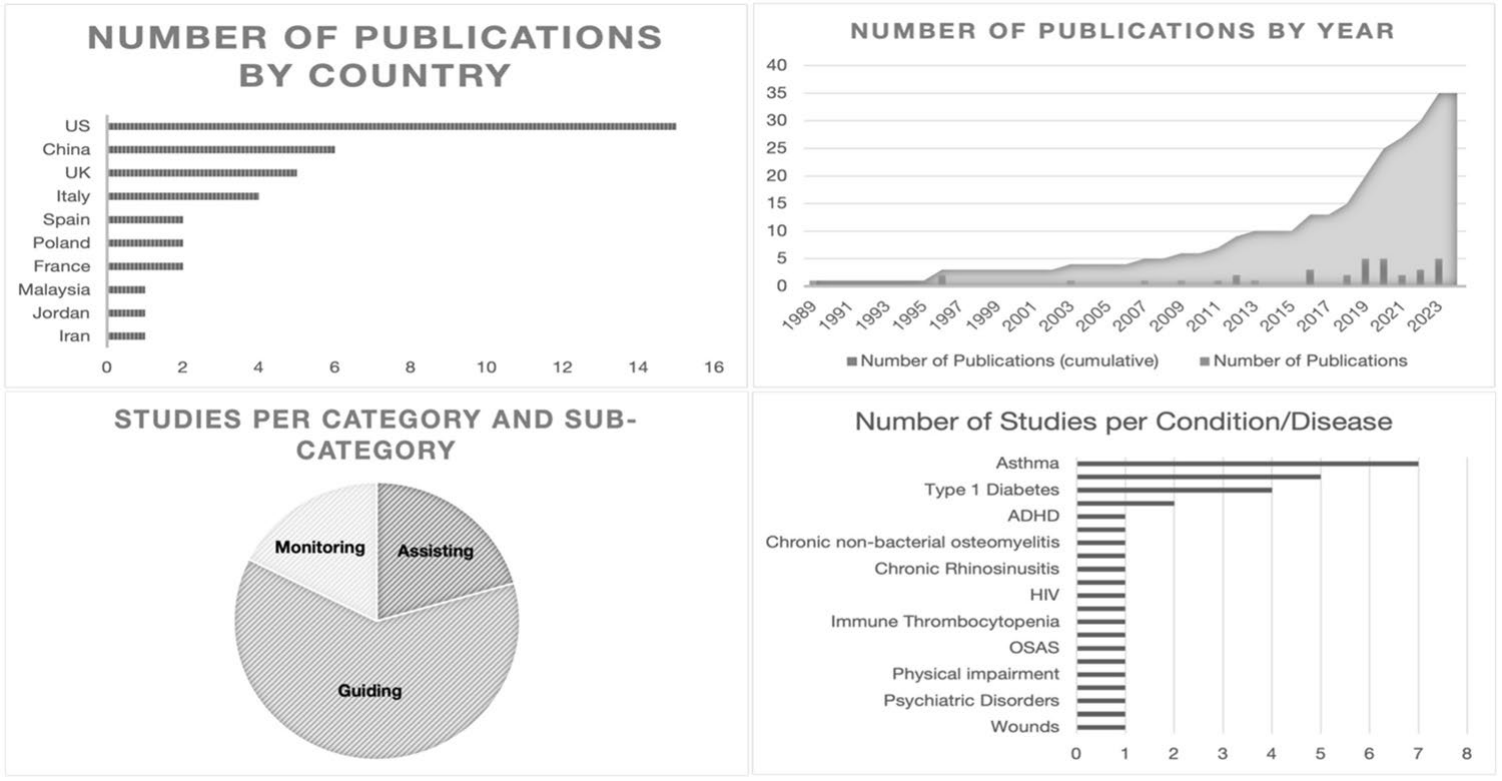
Characteristics of the included studies, depicting number of publications per country, year, category, and condition/disease

Our searches yielded 358 records; after removal of duplicates, 344 titles and abstracts were screened for eligibility out of which 111 full texts were examined. We identified 36 studies on the use of AI in monitoring, assisting, or guiding children and adolescents with chronic diseases (see Fig. 1). Most studies determined diagnostic accuracy for certain outcome parameters computed by an AI application, often compared to a gold standard or judgment by expert physicians. Level of implementation was generally low across the included studies (see Supplementary Information 1). Although many had been tested with real patient data—usually retrospective—no broad clinical application was described for any of the tools. Characteristics of included studies are shown in Fig. 2 and Table 1, Risk of Bias Assessments in Supplementary Information 3.

**Table 1:** Overview of AI methods used in the included studies: sub-categories, examples/algorithms, applications, and references. Studies were categorized into classical data science approaches and neural networks

Sub-category/method	Examples/algorithms	Application	References (examples)
**1. Classical data science approaches**			
1.1 Coded algorithms/conditionals/if–then-approaches	Rule-based conditional framework	Real-time monitoring applications (e.g., asthma monitoring)	[11, 12, 21, 28]
1.2 Classical machine learning statistics	Naive Bayes, Bayesian network, regressions, SVMs, Gaussian Processes, Decision Trees	Predictive modeling and diagnosis	[36, 37]
1.3 Ensemble learning methods	Random Forest, XGBoost, AdaBoost	Improving prediction accuracy by aggregating outputs of multiple models	[13, 17, 26, 37]
1.4 Case-based reasoning	Database of similar past cases	Real-time decision-making, controlling blood glucose levels in type 1 diabetes	[31]
**2. Neural networks**			
2.1 Convolutional Neural Networks (CNN)	Convolutional Neural Networks, scispaCy, Natural Language Processing	Interpretation of images (e.g., MRI scans), analyzing unstructured data from social media	[13, 16, 22]
2.2 Recurrent Neural Networks (RNN)	Recurrent Neural Networks, Long Short-Term Memory (LSTM)	Processing complex sequential data (e.g., electronic medical records), predicting disease progression	[10, 18, 23]

Most studies described tools for the use by healthcare professionals (classification, outcome prediction, and decision support tools) while there were few tools for the use by patients and their families [9–12]. Data from electronic health records and data generated specifically for the tool were most widely used.

### Monitoring children with chronic diseases

#### Classification of disease stages

Two studies used AI models for classification of diseases stages in individual patients. One compared classification of chronic kidney disease stages by different AI methods to an expert physician; the decision tree model performed best with 92% accuracy [13]; another study found that the developed algorithm was able to correctly classify asthma exacerbation in all tested cases [14].

#### Analysis of psychological distress and quality of life

A standardized questionnaire assessed Quality of Life (QoL) in individuals with irritable bowel disease (IBD). Utilizing AI for analysis, the study unveiled correlations between specific pre-existing conditions and psychological distress. Patients with elevated symptom levels were more prone to encountering psychological symptoms [15]. One article described a method using speech sampling of children with mental health issues to classify quality and intensity of emotions [16]. Two studies used social media data to evaluate psychological distress. One analyzed Facebook posts on diabetes support groups and found that the model performed best when taking not only the primary post into account, but also reactions from others [17]. The other found that a language-processing model was able to predict suicidal behavior from posts on the platform Reddit [18].

### Guiding children with chronic diseases

#### Prediction of disease outcomes

Several studies developed tools to predict the outcome or progression of a chronic disease. One study analyzed date from a cystic fibrosis registry [19]. The model was tested against real outcome data from the registry and performed well. Another model was able to accurately predict delayed serum creatinine decrease after kidney transplant [20]. One study identified markers for systemic inflammation as a key predictor for the need of dialysis in children with chronic kidney disease [21]. An algorithm to predict outcomes in patients with hypertension identified a set of variables and performed better than established outcome scores [22] and another was able to quickly identify wounds at risk for slow healing using quantitative data from a large dataset [23].

Three studies used asthma as a model disease to develop a tool predicting disease-related events; one identified risk factors for hospital readmission [24], another used a mix of patient and environmental factors to predict attacks [25]. Yu et al. developed a system that used multiple data sources both from patients and hospitals as well as various analysis methods to predict the absence or presence of health problems [26].

Other studies focused on predicting the development of secondary diseases or impairments related to a chronic disease or an injury. Two studies investigated outcomes after brain injury. One was able to predict outcomes [27], the other investigated the risk for attention deficit disorders [28]. In children with cerebral palsy, an algorithm found meaningful variables identifying those at risk of developing autism spectrum disorders [29]. An algorithm developed to predict development of chronic kidney disease after kidney injury was trained and performed well on retrospective data but performed poorly on prospective data [30]. The risk of chronicity for children with immune thrombocytopenia could be accurately predicted using a set of four AI models [31]. An algorithm for the early prediction of bronchopulmonal dysplasia in very low birthweight infants from a transcriptomic genetic signature performed well [32].

#### Prediction of therapy effectiveness

A model to predict improvement of hearing in patients with chronic otitis media after undergoing surgery made accurate predictions [33]. One in vitro study applied AI to predict the response of HIV positive patients to the influenza vaccine [34].

Image analysis techniques were used in two instances. One group developed an algorithm to assess effectiveness of treatment in children with chronic non-bacterial osteomyelitis and compared it to the rating by expert radiologists [35]. While the AI tool was able to detect all changes that had occurred over time, the overall accuracy evaluating success of treatment was lower than that of the radiologists. Another study used standardized photos to detect facial features predicting the persistence of an obstructive sleep apnea syndrome after surgery and identified several features [36].

#### Clinical decision support tools

Guidelines were used to provide support for asthma care in two studies; one developed a prototype concept that might help decide whether therapy should be (de-)escalated [37], the other used multiple data sources to make treatment suggestions to improve guideline adherence [38]. In a third study, a tool to estimate dry weight of children undergoing dialysis was developed and outperformed nephrologists [39].

### Assisting children with chronic diseases

#### Remote care and chronic disease management

An AI-based recommendation system for insulin bolus application was developed in an in silico experiment and tested with in silico cases against other systems, outperforming those with measurably more time in range [12].

Sendra et al. [11] developed a mobile application to aid children and adolescents with various chronic diseases; users were prompted to record data regarding their disease and therapy. Data were then reviewed by physicians and recommendations given to patients while an algorithm analyzed collected data as well as the recommendations. In a second iteration, recommendations were given by the AI tool which, after some training, were suitable for the situation. Similarly, another study used a data mining approach of individual data to monitor asthma and give automated recommendations based on expert knowledge [9]. Another in silico experiment proposed a method for structuring patient-generated health data to help identify symptoms and induce healthcare interventions [10].

#### Robotics

Robots as assistants were described for two different uses; Gosine et al. described the development of a prototype of an intelligent end-effector robot to aid physically impaired children using multiple sensors [40]. Two studies by an Italian-Dutch research group developed a humanoid robot to assist children and youth with type 1 diabetes [41, 42]. The robots were used in waiting rooms and summer camps. They played games with the children and helped calculate insulin doses.

### Acceptability of AI applications

Four studies evaluated the acceptance of AI applications by users. The abovementioned humanoid robots assisting children and adolescents with type 1 diabetes mellitus were perceived useful, accepted well as companions and children liked interacting with them; however, there was a notable difference in cultures as Italian children interacted more closely and personally with the robot and were more expressive verbally than Dutch children [41, 42]. Children also played games with the robot and accepted their mistakes. A mobile application to monitor children with chronic diseases was perceived as useful by parents and physicians [11]. A study investigating the attitude of clinicians towards AI-based interpretations of radiologic images found that they valued the systematic reporting, but trustworthiness was an issue. Clinicians generally put more trust into the findings if they aligned with their own or those of a trusted radiologist. They also put limited trust in the AI’s ability to report incidental findings [43].

### Performance and accuracy of AI

In most studies that reported prediction or classification accuracy, AI performed well compared to reference standards, expert judgments, or actual clinical data.

There were two instances in which AI did not perform as well; one was the classification of disease stages for chronic non-bacterial osteomyelitis based on MRI images in which AI only correctly classified a third. It was, however, able to identify improvement or worsening in all cases [35]. Morse et al. described the development of a model to predict chronic kidney disease after kidney injury and tested it prospectively on patients admitted to the hospital where it was not able to perform similarly well as it had previously with the retrospective training data set (AUROC 0.76 vs. 0.63) which is an important finding showing that performance on training data might not accurately predict the algorithm’s performance when deployed [30].

## Discussion

This review finds a high number of publications focusing on development of AI tools to aid children and adolescents with chronic diseases. Overall, however, AI these tools still play a minor role in guiding the care of chronically ill children and are often limited to parameters for which large amounts of data are readily available. Many studies were smaller proof-of-concept explorations. The observed poor performance with prospective clinical data after a successful trial with retrospective data in one study aligns well with other similar observations outside of pediatric applications, and poses the important question whether the promising results will hold true when applied broadly [30, 44].

One innovative approach is the analysis of social media data for early detection of mental health issues. This data is generated in an unstructured way by many users every day and is only analyzable with AI technology. The WHO emphasizes the role of digital technologies for improvement of mental health care especially in low-resource settings [45]. Our search yielded two examples, showing that social media data might be helpful for a holistic care. Mental health problems are an important comorbidity of primarily somatic chronic diseases and may lead to lower quality of life and poor social development and function [46]. This should be a focal point for further research and care. It has been shown that detecting mental health disorders through social media data is feasible and might be able to detect disorders earlier than conventional methods [47, 48]. Despite those potential benefits, privacy and the right to withhold certain information from parents or guardians need to be considered. Moreover, children and adolescents might use certain social codes in language that might lead to erroneous conclusions. Additionally, cultural, social, and gender factors might influence how one uses and expresses oneself on social media, making such analyses prone to discrimination.

A potentially very useful AI application that might guide children, parents, and healthcare professionals is a decision support tool that suggests treatment according to current guidelines aiming to improve physicians’ adherence to standardized recommendations [38]. As guideline adherence is generally optimizable [49], such tools could prove useful to ensure adequate treatment and patient safety especially for patients with rare diseases living far from a center specialized in that condition. An algorithm for the adaption of insulin bolus recommendation showed promising results and might further improve the rising technology of Automated Insulin Delivery (AID) systems [12, 50]. This might serve as an example for other conditions that need close monitoring of vital functions or blood levels and subsequent treatment decisions. Several studies investigated the outcome of different diseases after therapy and developed models to predict effectiveness [33–36] leading the way to personalized therapy decisions avoiding treatment that might be ineffective or harmful.

Trust and accountability are important concepts for the ethical use of AI and while AI is often viewed favorably and believed to play an important part in the future of care, errors, and responsibility are points of concern [51–53]. In pediatrics, patient autonomy is limited both for developmental and legal reasons. Parents or legal guardians play a crucial role in decision-making. Our search did not yield much evidence on parents’ attitudes in regard to children or adolescents with chronic diseases. A study with parents of healthy children [54] suggests a moderate openness towards AI-driven precision medicine although there are concerns regarding privacy. The lack of data investigating children’s and adolescents’ attitudes shows a need for research in this field. Whenever a broader application is planned, all stakeholders’ views should be considered.

Limitations of our study might stem from our search strategy. We aimed to gain a broad understanding of AI-based health care interventions. We included computer science databases, gray literature, and conference contributions. However, the use of English search terms may have led to the omission of evidence from non-English-speaking countries. Apart from India and China, there was a lack of studies from countries other than North America and Europe. Evidence from other countries might be especially interesting regarding feasibility of technology-driven healthcare interventions as well as attitudes which might be culturally driven. Our decision to include all studies regardless of their risk of bias led to a more comprehensive overview of research activities in the field. However, confidence in findings concerning accuracy of predictive tools might be limited.

This review offers an in-depth exploration of AI applications spanning the monitoring, guidance, and support of children and adolescents with chronic conditions. However, many studies are characterized by their limited scale and potential bias, with few having transitioned into genuine clinical practice. Consequently, further research and development are imperative, given the current restricted use of AI applications in the care of young patients with chronic illnesses. While its utilization remains largely confined to research endeavors, the growing domains of digital medicine and AI hint at a more extensive future role. The utility of AI is evident in targeted applications, but to progress, a comprehensive understanding of patient and healthcare provider perspectives is indispensable. To enhance the applicability of future studies, large-scale feasibility studies conducted in real clinical settings should be prioritized. These studies should evaluate not only the effectiveness of AI tools and data suitability but also their integration into existing healthcare workflows, facilitating applicability across diverse patient populations and different healthcare environments.

## Supplementary Information

Below is the link to the electronic supplementary material.

Supplementary file1 (XLSX 68 KB)

Supplementary file2 (DOCX 13 KB)

Supplementary file3 (PNG 2329 KB)

Supplementary file4 (PNG 475 KB)

Supplementary file5 (PNG 528 KB)

Supplementary file6 (PNG 447 KB)

Supplementary file7 (PNG 686 KB)

Supplementary file8 (PNG 473 KB)

## Data Availability

Data is provided within the supplementary information files as "Supplementary Information 1: Table of Included Studies".

## Abbreviations

AI: Artificial Intelligence
CBR: Case-Based Reasoning
CNN: Convolutional neural networks
LSTM: Long Short-Term Memory
ML: Machine Learning
PRISMA: Preferred Reporting Items for Systematic Reviews and Meta-Analyses
RNN: Recurrent neural networks
