# Supplementary material for: Artificial intelligence in the care of children and adolescents with chronic diseases: a systematic review
Source: Eur J Pediatr. 2024 Dec 14;184(1):83. doi: 10.1007/s00431-024-05846-3 (PMC11645428; doi:10.1007/s00431-024-05846-3)
Supplement: Supplementary file 2 — Supplementary file2 (DOCX 13 KB) [file 431_2024_5846_MOESM2_ESM.docx]

**Appendix XX Exemplary Search Strategy for PubMed/Medline**

Key Terms: Artificial Intelligence AND chronic disease AND children OR adolescents

MeSH Terms: (ai artificial intelligence[MeSH Terms]) AND (chronic disease[MeSH Terms]) AND ((children[MeSH Terms]) OR (adolescent[MeSH Terms]))

Search Strategy: ((ai artificial intelligence[MeSH Terms]) AND (chronic disease[MeSH Terms]) AND ((children[MeSH Terms]) OR (adolescent[MeSH Terms]))) OR (Artificial Intelligence AND chronic disease AND child* [All Fields] AND adolesc* [All Fields])
