# Supplementary figures and images for: Artificial intelligence in the care of children and adolescents with chronic diseases: a systematic review
Source: Eur J Pediatr. 2024 Dec 14;184(1):83. doi: 10.1007/s00431-024-05846-3 (PMC11645428; doi:10.1007/s00431-024-05846-3)

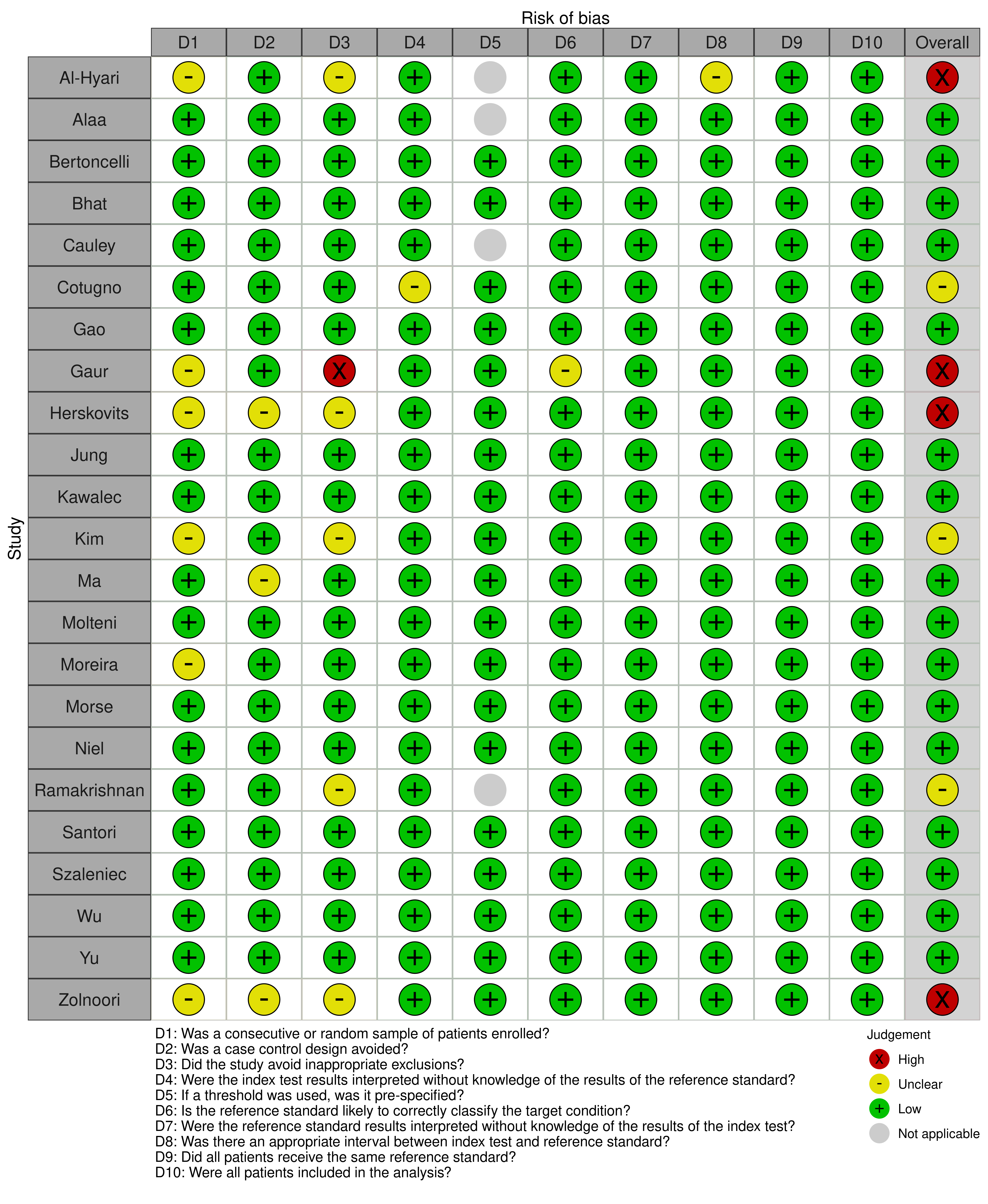

Supplement: Supplementary file 3 — Supplementary file3 (PNG 2329 KB) [file 431_2024_5846_MOESM3_ESM.png]

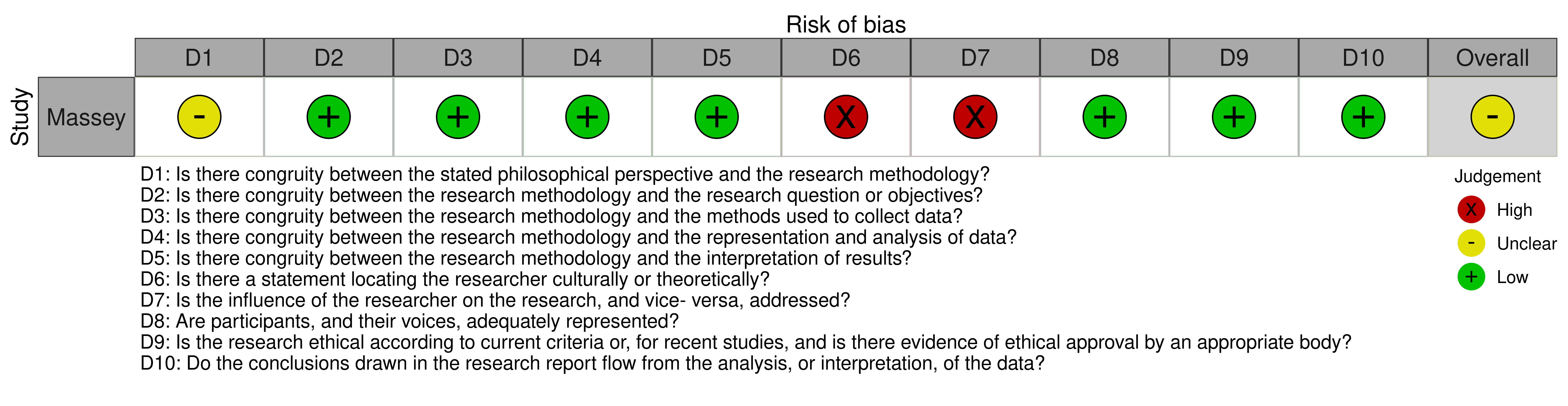

Supplement: Supplementary file 4 — Supplementary file4 (PNG 475 KB) [file 431_2024_5846_MOESM4_ESM.png]

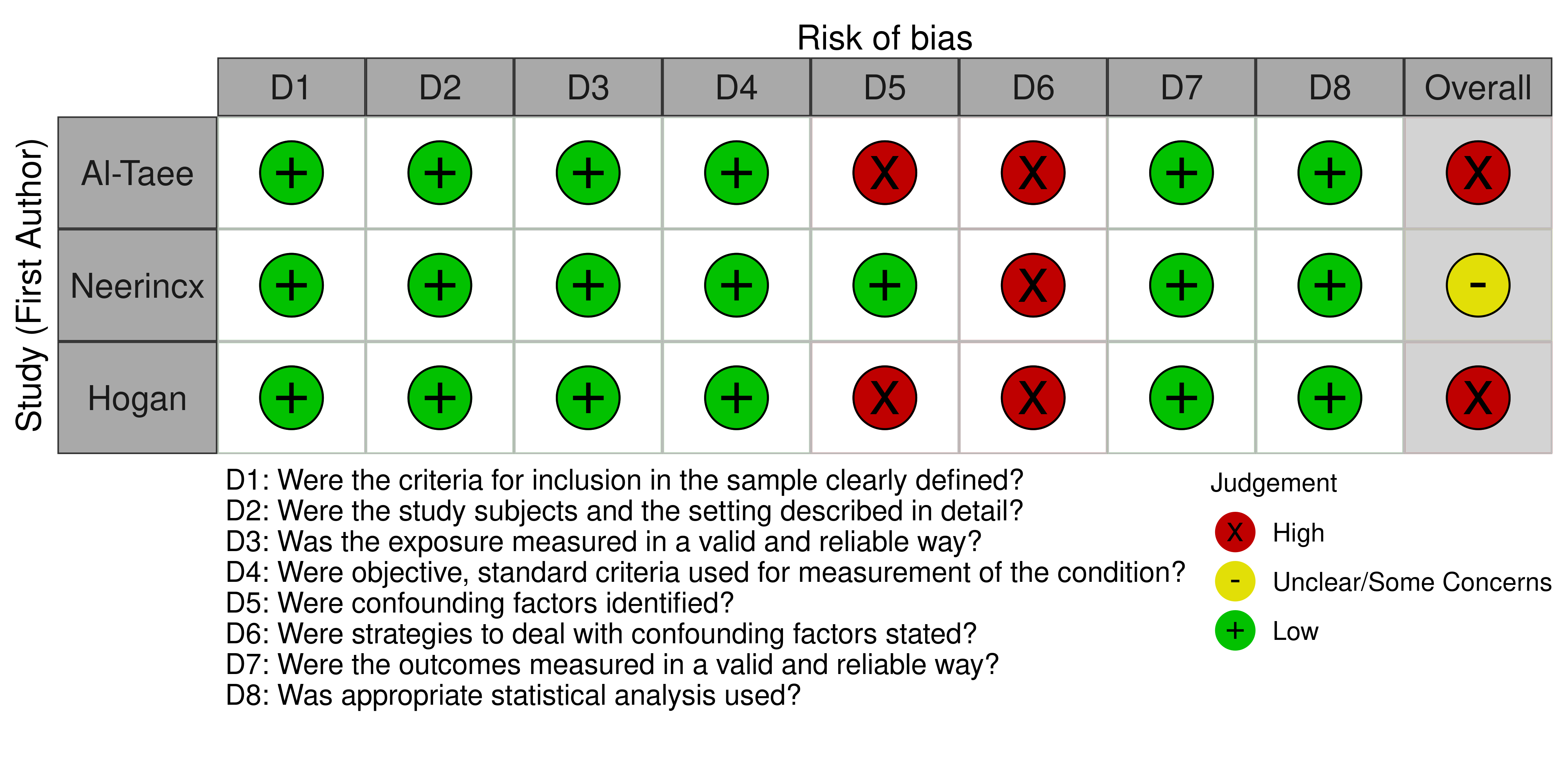

Supplement: Supplementary file 5 — Supplementary file5 (PNG 528 KB) [file 431_2024_5846_MOESM5_ESM.png]

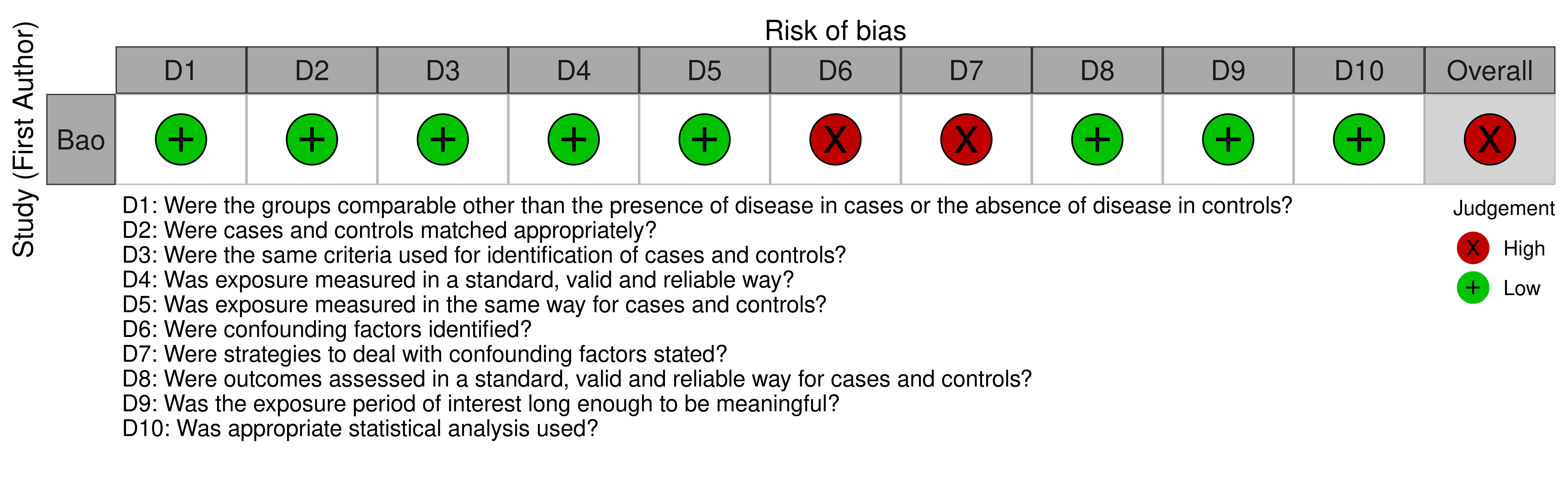

Supplement: Supplementary file 6 — Supplementary file6 (PNG 447 KB) [file 431_2024_5846_MOESM6_ESM.png]

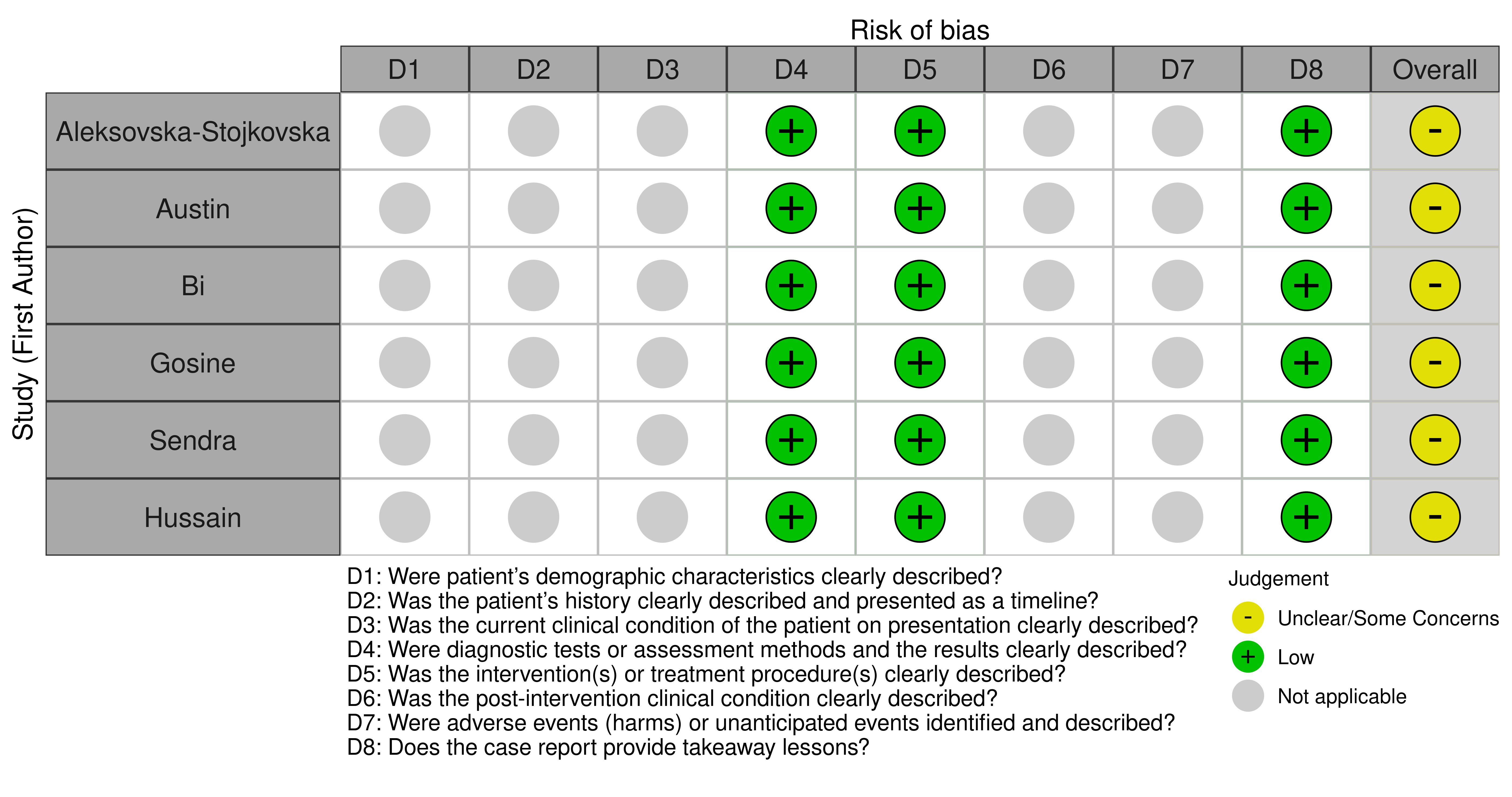

Supplement: Supplementary file 7 — Supplementary file7 (PNG 686 KB) [file 431_2024_5846_MOESM7_ESM.png]

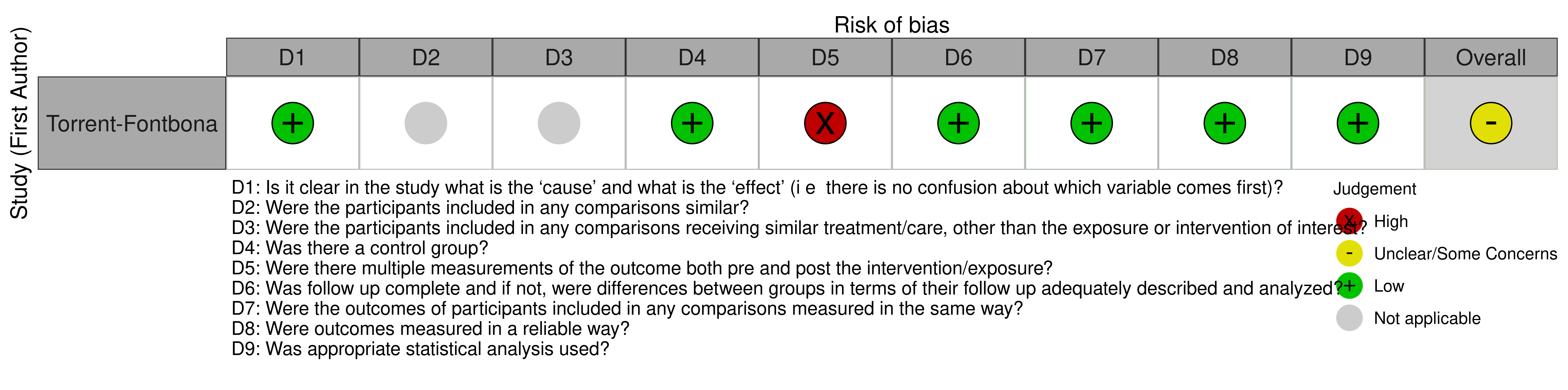

Supplement: Supplementary file 8 — Supplementary file8 (PNG 473 KB) [file 431_2024_5846_MOESM8_ESM.png]
